# PSYCHOSOCIAL FACTORS ASSOCIATED WITH SLEEP QUALITY AND DURATION AMONG OLDER ADULTS WITH CHRONIC PAIN

**DOI:** 10.1093/geroni/igz038.1935

**Published:** 2019-11-08

**Authors:** Catherine Zaidel, Shirley Musich, Jaycee Karl, Shaohung Wang, Sandra Kraemer, Charlotte Yeh

**Affiliations:** 1 Optum, Ann Arbor, Michigan, United States; 2 Optum, Minneapolis, Minnesota, United States; 3 UnitedHealthcare, Minneapolis, Minnesota, United States; 4 AARP Services, Inc., Washington, District of Columbia, United States

## Abstract

Sleep complaints are common among older adults with pain. Due to the risk of side effects, sleep medications are not recommended. Little is known about the association between psychosocial factors and sleep, but further awareness could support non-drug strategies for poor sleep. Our objective was to determine prevalence of self-reported poor sleep and duration among older adults with pain; and examine associations of positive psychosocial characteristics on sleep. This study analyzed surveys and claims from older adults with AARP® Medicare Supplement plans insured by UnitedHealthcare. Participants were 65+ with diagnosed back pain, osteoarthritis and/or rheumatoid arthritis; 12-months plan enrollment. All participants responded to a survey in May 2018 assessing sleep quality. Prescriptions were determined from claims. Propensity weighting was used to adjust for non-response bias. Results were weighted to generalize to those with pain. Multivariate logistic regression was used to evaluate associations. Short sleep duration was most common (39%), followed by poor quality (22%), and long duration (9%). Higher resilience and diverse social networks were associated with good quality and duration. Strongest associations with bad quality and short duration were stress, depression and sleep medications. Psychosocial factors were strongly associated with sleep quality and duration among older adults with pain. Results underscore the importance of social factors on sleep and need for non-drug sleep strategies.

